# 4-Hy­droxy-3-meth­oxy­benzaldehyde 4-phenyl­thio­semicarbazone

**DOI:** 10.1107/S1600536814002773

**Published:** 2014-02-12

**Authors:** Adriano Bof de Oliveira, Bárbara Regina Santos Feitosa, Christian Näther, Inke Jess

**Affiliations:** a Departamento de Química, Universidade Federal de Sergipe, Av. Marechal Rondon s/n, Campus, 49100-000 São Cristóvão, SE, Brazil; b Institut für Anorganische Chemie, Christian-Albrechts-Universität zu Kiel, Max-Eyth Strasse 2, D-24118 Kiel, Germany

## Abstract

In the title compound, C_15_H_15_N_3_O_2_S, the central C—N—N—C unit has an *anti* conformation [torsion angle = −170.17 (15)°]. The phenyl substituent is oriented perpendicular to this unit [dihedral angle of 89.2 (1)°], whereas the substituted ring is rotated out of this plane by only 18.86 (17)°. In the crystal, mol­ecules are linked by pairs of N—H⋯S hydrogen bonds into inversion dimers that are further connected *via* N—H⋯O and O—H⋯S hydrogen bonds into a three-dimensional network.

## Related literature   

For the synthesis and biological applications of thio­semicarbazone derivatives, see: Lovejoy & Richardson (2008 ▶). For one of the first reports on the synthesis of thio­semicarbazone derivatives, see: Freund & Schander (1902 ▶).
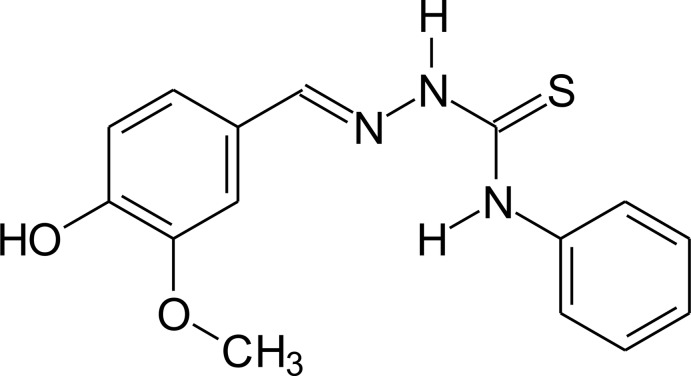


## Experimental   

### 

#### Crystal data  

C_15_H_15_N_3_O_2_S
*M* *_r_* = 301.36
Monoclinic, 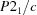

*a* = 11.1010 (5) Å
*b* = 8.7279 (4) Å
*c* = 15.7921 (7) Å
β = 105.008 (4)°
*V* = 1477.88 (12) Å^3^
*Z* = 4
Mo *K*α radiation
μ = 0.23 mm^−1^
*T* = 200 K
0.3 × 0.2 × 0.15 mm

#### Data collection  

Stoe IPDS-1 diffractometer
7883 measured reflections
2814 independent reflections
2401 reflections with *I* > 2σ(*I*)
*R*_int_ = 0.050

#### Refinement  

*R*[*F* ^2^ > 2σ(*F* ^2^)] = 0.034
*wR*(*F* ^2^) = 0.083
*S* = 1.04
2814 reflections
193 parameters
H-atom parameters constrained
Δρ_max_ = 0.22 e Å^−3^
Δρ_min_ = −0.18 e Å^−3^

### 

Data collection: *X-AREA* (Stoe & Cie, 2008 ▶); cell refinement: *X-AREA*; data reduction: *X-RED32* (Stoe & Cie, 2008 ▶); program(s) used to solve structure: *SHELXS97* (Sheldrick, 2008 ▶); program(s) used to refine structure: *SHELXL97* (Sheldrick, 2008 ▶); molecular graphics: *DIAMOND* (Brandenburg, 2006 ▶); software used to prepare material for publication: *publCIF* (Westrip, 2010 ▶).

## Supplementary Material

Crystal structure: contains datablock(s) I, publication_text. DOI: 10.1107/S1600536814002773/bt6961sup1.cif

Structure factors: contains datablock(s) I. DOI: 10.1107/S1600536814002773/bt6961Isup2.hkl

Supporting information file. DOI: 10.1107/S1600536814002773/bt6961Isup3.cml

CCDC reference:

Additional supporting information:  crystallographic information; 3D view; checkCIF report

## Figures and Tables

**Table 1:** Hydrogen-bond geometry (Å, °)

*D*—H⋯*A*	*D*—H	H⋯*A*	*D*⋯*A*	*D*—H⋯*A*
O1—H1*O*1⋯S1^i^	0.84	2.50	3.2844 (13)	157
N2—H1*N*2⋯S1^ii^	0.88	2.53	3.3460 (14)	155
N3—H1*N*3⋯O1^iii^	0.88	2.56	3.2068 (18)	131

Symmetry codes: (i) 
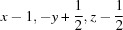
; (ii) 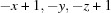; (iii) 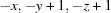.

**Fig. 1.**
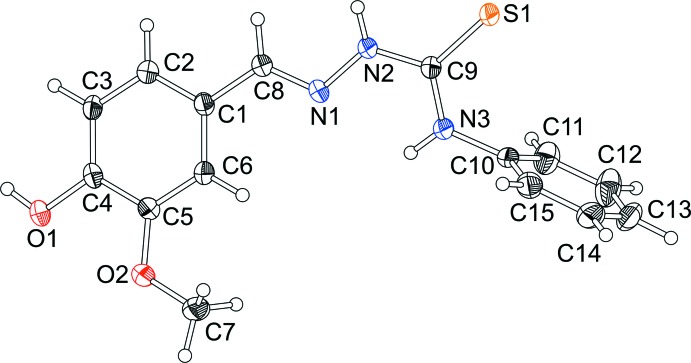
The molecular structure of the title compound with labeling and displacement ellipsoids drawn at the 40% probability level.

**Fig. 2.**
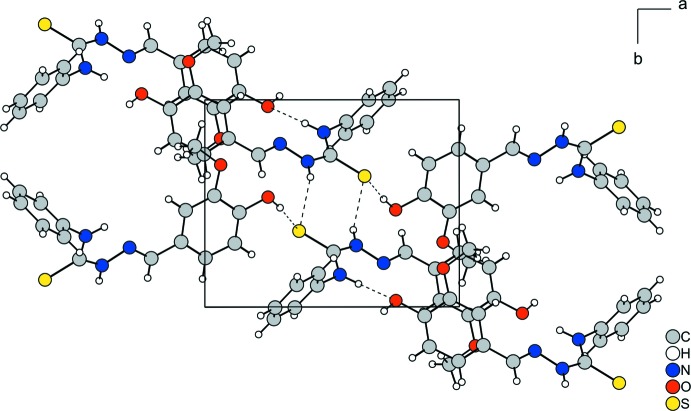
Part of the crystal structure of the title compound with view along the crystallographic c-axis. Intermolecular hydrogen bonding is shown as dashed lines.

## supplementary crystallographic information

### 1. Comment

The thiosemicarbazone chemistry has some impact on the search for new compounds used for the treatment of cancer. Thiosemicarbazone derivatives can act as ligands, *e.g.* with iron in the active centre of Fe-containing proteins and showing anti-proliferative activity against tumor cells (Lovejoy & Richardson, 2008). As part of our study on synthesis and structural chemistry of thiosemicarbazone derivatives, we report herein the crystal structure of a derivative of vanillin (4-Hydroxy-3-methoxybenzaldehyde).

In the crystal structure of the title compound the central CNNC unit is nearly planar with an torsion angle along C8—N1—N2—C9 of 170.17 (15)° and maximum deviations from the mean plane of 0.0542 (8) Å. The substituted phenyl ring (C1—C6) is slightly rotated out of this plane by 18.86 (17) °. In contrast, the unsubstituted phenyl ring (C10—C15) is perpendicular to the CNNC fragment with an dihedral angle of 89.2 (1)° (Fig. 1). The molecule shows a *trans* conformation about the C8—N1 and N1—N2 bonds.

In the crystal structure the molecules are linked by pairs of N—H···S hydrogen bonds into dimers that are located on centres of inversion (Fig. 2 and Table 1). These dimers are further linked by intermolecular N—H···O and O—H···S hydrogen bonding into a three-dimensional hydrogen bonded network (Fig. 2 and Table 1).

### 2. Experimental

Starting materials were commercially available and were used without further purification. The title compound synthesis was adapted from a procedure reported previously (Freund & Schander, 1902). The hydrochloric acid catalyzed reaction of vanillin (8,83 mmol) and 4-phenylthiosemicarbazide (8,83 mmol) in ethanol (50 ml) was refluxed for 6 h. After cooling and filtering, the title compound was obtained. Crystals suitable for X-ray diffraction were obtained in ethanol by the slow evaporation of solvent.

### 3. Refinement

All non-hydrogen atoms were refined anisotropic. All H atoms were located in difference map but were positioned with idealized geometry (methyl and O—H H atoms allowed to rotate but no to tip) and were refined isotropic with *U*_iso_(H) = 1.2 *U*_eq_(C, N,*O*) (1.5 for methyl and O—H H atoms) using a riding model with C—H = 0.95 Å for aromatic, C—H = 0.98 Å for methyl, N—H = 0.88 Å for amine and hydrazine O—H = 0.84 Å for hydroxyl H atoms.

### Crystal data

**Table:**

C_15_H_15_N_3_O_2_S	*F*(000) = 632
*M**_r_* = 301.36	*D*_x_ = 1.354 Mg m^−^^3^
Monoclinic, *P*2_1_/*c*	Mo *K*α radiation, λ = 0.71073 Å
Hall symbol: -P 2ybc	Cell parameters from 2916 reflections
*a* = 11.1010 (5) Å	θ = 1.9–26.0°
*b* = 8.7279 (4) Å	µ = 0.23 mm^−^^1^
*c* = 15.7921 (7) Å	*T* = 200 K
β = 105.008 (4)°	Plate, yellow
*V* = 1477.88 (12) Å^3^	0.3 × 0.2 × 0.15 mm
*Z* = 4	

### Data collection

**Table:**

Stoe IPDS-1 diffractometer	2401 reflections with *I* > 2σ(*I*)
Radiation source: fine-focus sealed tube, Stoe IPDS-1	*R*_int_ = 0.050
Graphite monochromator	θ_max_ = 26.0°, θ_min_ = 1.9°
φ scans	*h* = −13→13
7883 measured reflections	*k* = −10→10
2814 independent reflections	*l* = −19→16

### Refinement

**Table:**

Refinement on *F*^2^	Secondary atom site location: difference Fourier map
Least-squares matrix: full	Hydrogen site location: inferred from neighbouring sites
*R*[*F*^2^ > 2σ(*F*^2^)] = 0.034	H-atom parameters constrained
*wR*(*F*^2^) = 0.083	*w* = 1/[σ^2^(*F*_o_^2^) + (0.0283*P*)^2^ + 0.5666*P*] where *P* = (*F*_o_^2^ + 2*F*_c_^2^)/3
*S* = 1.04	(Δ/σ)_max_ = 0.001
2814 reflections	Δρ_max_ = 0.22 e Å^−^^3^
193 parameters	Δρ_min_ = −0.18 e Å^−^^3^
0 restraints	Extinction correction: *SHELXL97* (Sheldrick, 2008), Fc^*^=kFc[1+0.001xFc^2^λ^3^/sin(2θ)]^-1/4^
Primary atom site location: structure-invariant direct methods	Extinction coefficient: 0.0051 (19)

### Special details

**Table:**

Geometry. All e.s.d.'s (except the e.s.d. in the dihedral angle between two l.s. planes) are estimated using the full covariance matrix. The cell e.s.d.'s are taken into account individually in the estimation of e.s.d.'s in distances, angles and torsion angles; correlations between e.s.d.'s in cell parameters are only used when they are defined by crystal symmetry. An approximate (isotropic) treatment of cell e.s.d.'s is used for estimating e.s.d.'s involving l.s. planes.
Refinement. Refinement of *F*^2^ against ALL reflections. The weighted *R*-factor *wR* and goodness of fit *S* are based on *F*^2^, conventional *R*-factors *R* are based on *F*, with *F* set to zero for negative *F*^2^. The threshold expression of *F*^2^ > σ(*F*^2^) is used only for calculating *R*-factors(gt) *etc*. and is not relevant to the choice of reflections for refinement. *R*-factors based on *F*^2^ are statistically about twice as large as those based on *F*, and *R*- factors based on ALL data will be even larger.

### Fractional atomic coordinates and isotropic or equivalent isotropic displacement parameters (Å^2^)

**Table:**

	*x*	*y*	*z*	*U*_iso_*/*U*_eq_	
C1	0.09380 (14)	0.31502 (18)	0.38433 (10)	0.0277 (3)	
C2	−0.00285 (14)	0.23811 (19)	0.32665 (11)	0.0312 (4)	
H2	0.0098	0.1369	0.3085	0.037*	
C3	−0.11786 (14)	0.30837 (19)	0.29539 (11)	0.0308 (4)	
H3	−0.1842	0.2543	0.2569	0.037*	
C4	−0.13632 (14)	0.45689 (19)	0.31995 (10)	0.0289 (3)	
C5	−0.03748 (14)	0.53832 (18)	0.37529 (10)	0.0277 (3)	
C6	0.07610 (14)	0.46702 (18)	0.40785 (10)	0.0279 (3)	
H6	0.1426	0.5209	0.4463	0.033*	
O1	−0.24864 (10)	0.53058 (14)	0.29167 (8)	0.0384 (3)	
H1O1	−0.2957	0.4796	0.2513	0.058*	
O2	−0.06289 (10)	0.68653 (13)	0.39208 (8)	0.0354 (3)	
C7	0.03941 (16)	0.7771 (2)	0.43923 (13)	0.0408 (4)	
H7A	0.0712	0.7353	0.4984	0.061*	
H7B	0.0116	0.8829	0.4430	0.061*	
H7C	0.1058	0.7754	0.4087	0.061*	
C8	0.21140 (14)	0.23577 (18)	0.41893 (11)	0.0298 (4)	
H8	0.2254	0.1416	0.3928	0.036*	
N1	0.29680 (11)	0.28779 (15)	0.48318 (9)	0.0283 (3)	
N2	0.40487 (11)	0.20239 (15)	0.50512 (9)	0.0292 (3)	
H1N2	0.4160	0.1276	0.4706	0.035*	
C9	0.49347 (13)	0.23299 (17)	0.57920 (10)	0.0262 (3)	
S1	0.62845 (4)	0.13258 (5)	0.60149 (3)	0.03137 (14)	
N3	0.46941 (12)	0.34351 (16)	0.63052 (9)	0.0319 (3)	
H1N3	0.3955	0.3873	0.6159	0.038*	
C10	0.55817 (14)	0.39469 (17)	0.70857 (11)	0.0293 (4)	
C11	0.55360 (17)	0.3387 (3)	0.78865 (12)	0.0442 (5)	
H11	0.4933	0.2639	0.7929	0.053*	
C12	0.63836 (19)	0.3927 (3)	0.86368 (13)	0.0574 (6)	
H12	0.6370	0.3536	0.9196	0.069*	
C13	0.72416 (17)	0.5025 (3)	0.85702 (14)	0.0530 (6)	
H13	0.7806	0.5408	0.9085	0.064*	
C14	0.72884 (18)	0.5569 (2)	0.77684 (15)	0.0501 (5)	
H14	0.7890	0.6319	0.7727	0.060*	
C15	0.64570 (16)	0.5026 (2)	0.70154 (13)	0.0407 (4)	
H15	0.6490	0.5395	0.6456	0.049*	

### Atomic displacement parameters (Å^2^)

**Table:**

	*U*^11^	*U*^22^	*U*^33^	*U*^12^	*U*^13^	*U*^23^
C1	0.0247 (7)	0.0305 (8)	0.0259 (8)	0.0031 (6)	0.0030 (6)	0.0008 (6)
C2	0.0306 (8)	0.0301 (8)	0.0297 (9)	0.0020 (6)	0.0021 (7)	−0.0022 (6)
C3	0.0257 (7)	0.0330 (8)	0.0285 (8)	−0.0013 (6)	−0.0025 (6)	−0.0008 (7)
C4	0.0228 (7)	0.0344 (8)	0.0273 (8)	0.0037 (6)	0.0022 (6)	0.0037 (6)
C5	0.0269 (7)	0.0271 (8)	0.0282 (8)	0.0029 (6)	0.0057 (6)	0.0012 (6)
C6	0.0236 (7)	0.0303 (8)	0.0272 (8)	0.0002 (6)	0.0022 (6)	−0.0011 (6)
O1	0.0251 (6)	0.0409 (7)	0.0421 (7)	0.0077 (5)	−0.0044 (5)	−0.0037 (5)
O2	0.0308 (6)	0.0288 (6)	0.0421 (7)	0.0050 (5)	0.0011 (5)	−0.0036 (5)
C7	0.0380 (9)	0.0296 (8)	0.0518 (12)	−0.0023 (7)	0.0061 (8)	−0.0067 (8)
C8	0.0269 (7)	0.0291 (8)	0.0301 (9)	0.0040 (6)	0.0014 (7)	−0.0020 (6)
N1	0.0238 (6)	0.0286 (7)	0.0295 (7)	0.0053 (5)	0.0017 (5)	0.0014 (5)
N2	0.0243 (6)	0.0300 (7)	0.0292 (7)	0.0068 (5)	−0.0002 (5)	−0.0053 (6)
C9	0.0245 (7)	0.0266 (7)	0.0253 (8)	0.0002 (6)	0.0025 (6)	−0.0005 (6)
S1	0.0239 (2)	0.0357 (2)	0.0300 (2)	0.00726 (16)	−0.00104 (15)	−0.00724 (17)
N3	0.0249 (6)	0.0327 (7)	0.0334 (8)	0.0063 (5)	−0.0010 (6)	−0.0083 (6)
C10	0.0263 (7)	0.0275 (8)	0.0315 (9)	0.0047 (6)	0.0029 (6)	−0.0075 (6)
C11	0.0332 (9)	0.0618 (12)	0.0379 (10)	−0.0056 (8)	0.0099 (8)	−0.0032 (9)
C12	0.0436 (11)	0.0995 (18)	0.0290 (10)	0.0041 (12)	0.0093 (9)	−0.0088 (11)
C13	0.0331 (9)	0.0692 (14)	0.0500 (13)	0.0050 (9)	−0.0013 (9)	−0.0333 (11)
C14	0.0405 (10)	0.0360 (10)	0.0659 (14)	−0.0051 (8)	−0.0002 (10)	−0.0125 (9)
C15	0.0405 (9)	0.0313 (9)	0.0461 (11)	−0.0028 (7)	0.0034 (8)	0.0023 (8)

### Geometric parameters (Å, º)

**Table:**

C1—C2	1.387 (2)	N1—N2	1.3783 (17)
C1—C6	1.405 (2)	N2—C9	1.3458 (19)
C1—C8	1.453 (2)	N2—H1N2	0.8800
C2—C3	1.387 (2)	C9—N3	1.331 (2)
C2—H2	0.9500	C9—S1	1.6924 (15)
C3—C4	1.383 (2)	N3—C10	1.4356 (19)
C3—H3	0.9500	N3—H1N3	0.8800
C4—O1	1.3710 (18)	C10—C11	1.369 (3)
C4—C5	1.406 (2)	C10—C15	1.378 (2)
C5—O2	1.3645 (19)	C11—C12	1.390 (3)
C5—C6	1.380 (2)	C11—H11	0.9500
C6—H6	0.9500	C12—C13	1.375 (3)
O1—H1O1	0.8400	C12—H12	0.9500
O2—C7	1.425 (2)	C13—C14	1.366 (3)
C7—H7A	0.9800	C13—H13	0.9500
C7—H7B	0.9800	C14—C15	1.386 (3)
C7—H7C	0.9800	C14—H14	0.9500
C8—N1	1.2792 (19)	C15—H15	0.9500
C8—H8	0.9500		
			
C2—C1—C6	119.54 (14)	C8—N1—N2	115.18 (13)
C2—C1—C8	118.90 (14)	C9—N2—N1	120.24 (13)
C6—C1—C8	121.57 (14)	C9—N2—H1N2	119.9
C3—C2—C1	120.26 (15)	N1—N2—H1N2	119.9
C3—C2—H2	119.9	N3—C9—N2	117.17 (13)
C1—C2—H2	119.9	N3—C9—S1	123.73 (11)
C4—C3—C2	120.26 (14)	N2—C9—S1	119.10 (12)
C4—C3—H3	119.9	C9—N3—C10	123.17 (13)
C2—C3—H3	119.9	C9—N3—H1N3	118.4
O1—C4—C3	122.45 (14)	C10—N3—H1N3	118.4
O1—C4—C5	117.57 (14)	C11—C10—C15	120.93 (16)
C3—C4—C5	119.98 (14)	C11—C10—N3	120.05 (15)
O2—C5—C6	124.74 (14)	C15—C10—N3	119.01 (16)
O2—C5—C4	115.64 (13)	C10—C11—C12	119.19 (19)
C6—C5—C4	119.62 (14)	C10—C11—H11	120.4
C5—C6—C1	120.26 (14)	C12—C11—H11	120.4
C5—C6—H6	119.9	C13—C12—C11	120.0 (2)
C1—C6—H6	119.9	C13—C12—H12	120.0
C4—O1—H1O1	109.5	C11—C12—H12	120.0
C5—O2—C7	116.78 (12)	C14—C13—C12	120.46 (18)
O2—C7—H7A	109.5	C14—C13—H13	119.8
O2—C7—H7B	109.5	C12—C13—H13	119.8
H7A—C7—H7B	109.5	C13—C14—C15	119.98 (19)
O2—C7—H7C	109.5	C13—C14—H14	120.0
H7A—C7—H7C	109.5	C15—C14—H14	120.0
H7B—C7—H7C	109.5	C10—C15—C14	119.41 (19)
N1—C8—C1	122.57 (15)	C10—C15—H15	120.3
N1—C8—H8	118.7	C14—C15—H15	120.3
C1—C8—H8	118.7		
			
C6—C1—C2—C3	−2.8 (3)	C1—C8—N1—N2	−177.00 (14)
C8—C1—C2—C3	177.29 (16)	C8—N1—N2—C9	−170.17 (15)
C1—C2—C3—C4	1.4 (3)	N1—N2—C9—N3	2.5 (2)
C2—C3—C4—O1	−179.19 (16)	N1—N2—C9—S1	−176.66 (11)
C2—C3—C4—C5	1.4 (3)	N2—C9—N3—C10	−176.04 (15)
O1—C4—C5—O2	−2.8 (2)	S1—C9—N3—C10	3.1 (2)
C3—C4—C5—O2	176.67 (15)	C9—N3—C10—C11	−97.2 (2)
O1—C4—C5—C6	177.76 (15)	C9—N3—C10—C15	84.1 (2)
C3—C4—C5—C6	−2.8 (2)	C15—C10—C11—C12	0.3 (3)
O2—C5—C6—C1	−178.00 (16)	N3—C10—C11—C12	−178.35 (17)
C4—C5—C6—C1	1.4 (2)	C10—C11—C12—C13	1.0 (3)
C2—C1—C6—C5	1.4 (2)	C11—C12—C13—C14	−1.5 (3)
C8—C1—C6—C5	−178.71 (16)	C12—C13—C14—C15	0.8 (3)
C6—C5—O2—C7	6.7 (2)	C11—C10—C15—C14	−1.1 (3)
C4—C5—O2—C7	−172.72 (15)	N3—C10—C15—C14	177.63 (16)
C2—C1—C8—N1	−167.58 (16)	C13—C14—C15—C10	0.5 (3)
C6—C1—C8—N1	12.5 (3)		

### Hydrogen-bond geometry (Å, º)

**Table:**

*D*—H···*A*	*D*—H	H···*A*	*D*···*A*	*D*—H···*A*
O1—H1*O*1···S1^i^	0.84	2.50	3.2844 (13)	157
N2—H1*N*2···S1^ii^	0.88	2.53	3.3460 (14)	155
N3—H1*N*3···O1^iii^	0.88	2.56	3.2068 (18)	131

Symmetry codes: (i) *x*−1, −*y*+1/2, *z*−1/2; (ii) −*x*+1, −*y*, −*z*+1; (iii) −*x*, −*y*+1, −*z*+1.
